# Residual malformations and leg length discrepancy after treatment of fibular hemimelia

**DOI:** 10.1186/1749-799X-6-51

**Published:** 2011-09-27

**Authors:** Dimosthenis A Alaseirlis, Anastasios V Korompilias, Alexandros E Beris, Panayotis N Soucacos

**Affiliations:** 1 Department of Orthopaedic Surgery, General Hospital of Giannitsa, Terma Semertzidi Str., 58100, Giannitsa, Greece; 2 Department of Orthopaedic Surgery, University Hospital of Ioannina, Stavros Niarchos Avenue, 45500, Ioannina, Greece; 3 1st Department of Orthopaedic Surgery, School of Medicine, University of Athens, "Attikon" Hospital, Rimini 1 Haidari 12462, Athens, Greece

## Abstract

**Background:**

Fibular hemimelia has been reported as the most common congenital longitudinal deficiency of the long bones. Previous studies have focused on the best treatment options for this congenital condition. There is very little to our knowledge in the literature focused on residual persisting malformations and leg length discrepancy after treatment.

**Methods:**

Seven patients presenting fibular hemimelia in eight fibulae received treatment between years 1988 and 2001. Pre-treatment average leg length discrepancy was 5.3 cm. All patients presented associated congenital deformities of the ipsilateral leg. Six patients received surgical treatment. Average post-treatment follow up was 9.7 years. Residual malformations and leg length discrepancy were recorded for all patients. It is a retrospective case series study at one institution by two of the presenting authors as senior surgeons.

**Results:**

Average leg length gained after successful bone lengthening in six patients was 5.06 cm. Although there was a significant functional improvement, a number of residual malformations and leg length inequality was recorded. Residual average leg length discrepancy of 3.1 cm was observed in five patients who completed surgical treatment. Five patients presented a limp. Residual anterior-medial bowing of the tibia was observed in four patients. Calf atrophy was present in all seven patients. Valgus deformity of the ankle was remained in two patients.

**Conclusions:**

Treatment of fibular hemimelia, even in cases graded as successful, showed to be accompanied by a number of persisting residual deformities and recurrent leg length inequality. Although the number of patients is limited, the high rate of this phenomenon is indicative of the significance of the report. The family and the patients themselves should have the right expectations and will be more co-operative when well informed about this instance. A report of common post-treatment residual deformities should be valuable in best possible treatment planning of fibular hemimelia.

## Background

Fibular hemimelia has been reported as the most common longitudinal congenital deficiency of the long bones [1,2]. Pathological involvement of the entire lower extremity very often associates this congenital condition. Femur hypoplasia, anterior and medial angulation of the tibia, valgus deformity and instability of the knee and ankle joints, and absence of the lateral foot rays are the most common congenital malformations that co-exist with fibular hemimelia [3]. There is a considerable number of previous studies focused on the best treatment options regarding the choice between lengthening, epiphysiodesis or amputation, the correction of leg malalignement and the choice of the most appropriate devices and surgical techniques. Despite the better understanding of the natural history of fibular hemimelia and the improvements in surgical techniques and devices, we still have to face the problem of residual malformations and leg length discrepancy at the completion of the treatment, even when it has been graded as successful. Joint instability, leg axis malalignement, persisting angulation of the tibia and leg length discrepancy have been occasionally reported to remain or recur after treatment [4-6].

There are very few studies in our knowledge, focusing on the residual malformations and discrepancy after treatment of fibular hemimelia.

The purpose of this study is to report the incidence and characteristics of residual problems and persisting malformations after treatment of fibular hemimelia, and to examine how much disabling these problems really are.

## Methods

It is a retrospective case series study at one institution by two of the presenting authors as senior surgeons. Between years 1988 and 2001, ten patients were referred to our Department and received treatment for fibular hemimelia. Three patients excluded from the study as they did not met the criteria of a complete follow-up. Finally, seven patients presenting fibular hemimelia in eight fibulae included in the present study. Three patients were male and four were female. Mean age on first examination was 4 years and 2.4 months old (ranged from three days to nine years old).

Details of the patients and types of treatment are presented in Additional File 1, Table S1. No known syndromes were detected. Six of seven patients were treated surgically presenting average leg length discrepancy (LLD) of 5.3 cm (ranged 2.9 to 9.0 cm) preoperatively. The average projected LLD at skeletal maturity was evaluated to be 9.1 cm, combining all the available methods: the Green-Anderson charts and the Menelaus method in all patients, where as the Moseley graph was used selectively in three patients (patients 1, 2 and 3) [Additional file 1, table s1] who were followed for more than four years before surgical treatment [7-9]. All patients underwent CT topograms on first presence followed by repeated measurements in annual basis for accurate evaluation of current and projected LLD. In all patients with LLD there was even minimal contribution of femur hypoplasia to leg length inequality, as it was measured using CT topograms.

Fifteen totally procedures included in the initial treatment plan: five lengthening procedures of the tibia, two of the femur, four of the Achilles, two of the peroneal tendons, one corrective osteotomy of the tibia, one corrective osteotomy and arthrodesis of the tarsus [Additional file 1, table s1]. All patients received primary lengthening procedures in our department by two of the presenting authors expertised in this field (A.E.B and P.N.S.). LLD of more than 2.5 cm was initially planned to be treated by lengthening procedures. Average preoperative follow up was 3 years and 8 months (1 to 7 years).

LLD was treated by lengthening of the tibia alone in three patients (cases 1, 4, 6). Lengthening of both the tibia and femur in two different sequential time points was required in two patients (cases 2, 7). Average age on first lengthening procedure was 7.6 years (ranged 3 to 15). Average pre-lengthening follow-up to our center was 6.6 years (ranged 2 to 10). Monolateral fixator devices were used in all primary lengthening procedures. Lengthening of the tibia or of the femur was canceled during growth spur periods to avoid unpredictable results. Lengthening rate was 1 mm per day. Radiographic examination was routinely followed until the end of consolidation phase.

Leg length was evaluated postoperatively using CT topograms on 12 months intervals. Last measurement of leg length was made in the mean age of 15 years old (13 to 20) in four of the five patients who underwent lengthening procedures, depending on clinical and radiographic findings of the rate of LLD reoccurrence and the age of skeletal maturity of each patient. Last topogram was made in exception in patient 4 [Additional file 1, table s1], [Additional file 2, table s2] in the age of 8 years old, as there was no need for further evaluation of leg length beyond this age.

Additional lengthening of the tibia as a revision procedure was needed in patients 4, 6 and 7, additional lengthening of the femur in patient 7, two revision corrective osteotomies of the tibia in patient 6 and a revision achilles lengthening procedure in patient 6. Totally seven revision procedures were needed, with two totally lengthening procedures of the tibia in patient 4, two lengthening procedures of the Achilles, two lengthening procedures and three corrective osteotomies of the tibia in patient 6 and two lengthening procedures of the femur and of the tibia in patient 7. Circular fixator devices were used in all instances of additional lengthenings and osteotomies of the involved bones. All revision procedures were done in our Department by the two senior surgeons already mentioned. Average post-treatment follow up was 9.7 years (1 to 18 years). Functional outcome is shown in details [Additional file 3, table s3], and was evaluated according to Lower Extremity Functional Scale (LEFS) [10].

## Results

Average leg length gained after successful bone lengthening was 5.06 cm (ranged 4 to 6). Consolidation phase was proved to be 4.67 times the lengthening time, resulting in 54.9 days/cm average healing index [11]. Conservative treatment modalities either as permanent (cast in patient 5) or as temporary treatment (shoe elevation in patients 1 and 3) proved to be successful. Average post-treatment LEFS after 9.7 years follow-up was 89.4% (ranged 53% to 100%) compared to average pretreatment LEFS which was 70.9% (ranged 53% to 96%).

After treatment there was even minimal LLD in five of six patients who were treated surgically, with 4.08 cm being the average value (ranged 0 to 9 cm) in all five patients as shown in Additional File 2, Table S2. Residual LLD of 2.0 cm was finally recorded in the one patient that treated conservatively. The final LLD was recorded at both the end of treatment and at the last follow-up in three patients (patients 1, 4 and 5), at the last follow-up in three patients (2, 3 and 7) who are suggested to be also considered as measured at the end of treatment while these patients do not wish a new lengthening procedure and at the last follow-up in one patient (patient 6) who wish a new lengthening procedure combined with a revision corrective osteotomy of the tibia.

**Figure 1:**
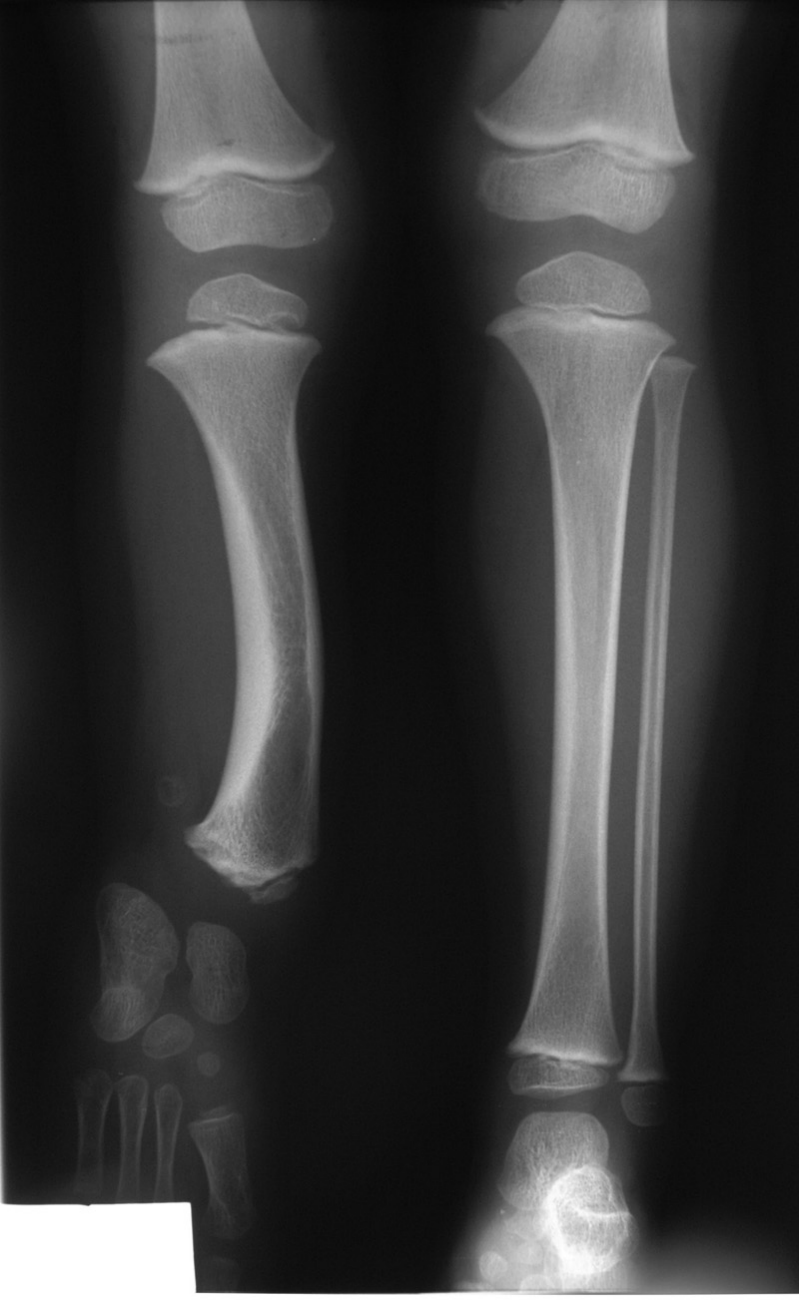
**Radiographic presentation of a 6 years old patient with fibular hemimelia type II**. Patient's (case 6) radiographs on first examination in the age of 6 years old showing a complete absence of the fibula, antero-medial bowing of the tibia which is significantly shorter compared to the contralateral one and dysplasia of the lateral distal epiphysis of the tibia.

**Figure 2:**
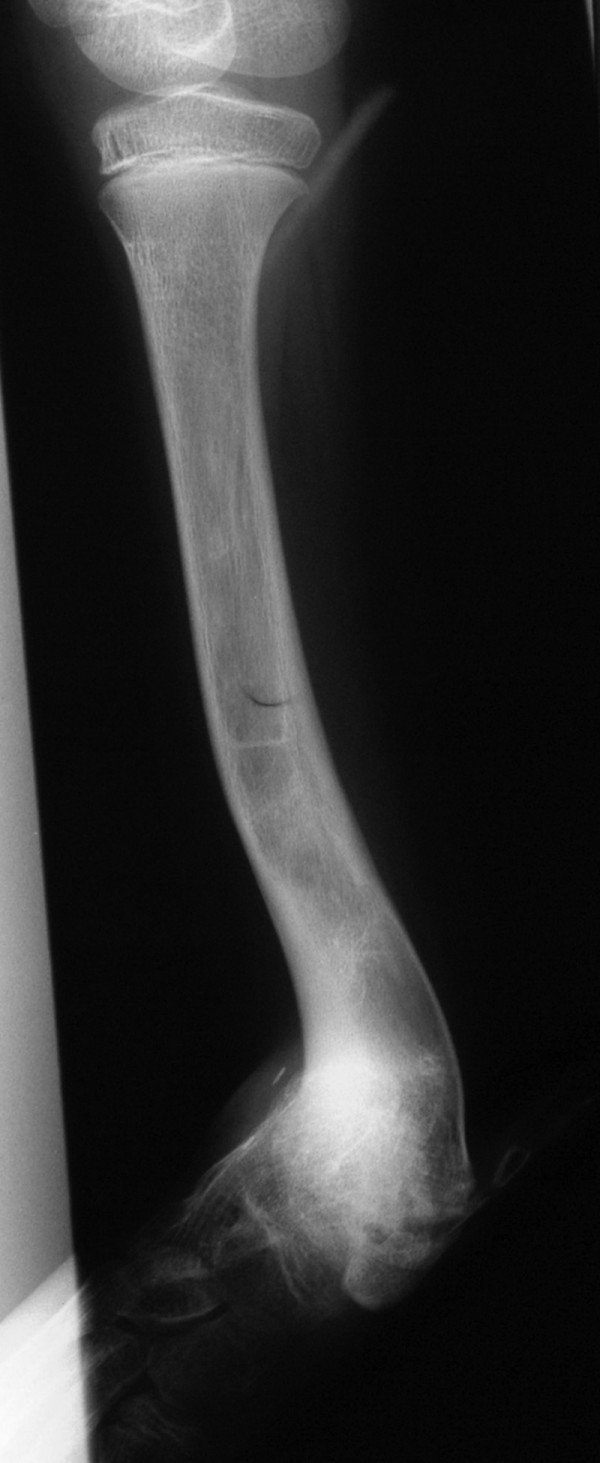
**Radiographic presentation of the same patient two years after lengthening procedure**. Severe valgus deformity of 85° of the distal tibia in the age of nine y.o., and two years after completing a lengthening procedure of the tibia. Despite multiple efforts to correct axial malignment during lengthening this was not gained and a severe valgus deformity of the ankle with a prominent antero-medial bowing of the tibia were obvious. It is believed that a severe dysplasia of the lateral distal epiphysis of the tibia is mainly responsible for this resisting to treatment deformity.

**Figure 3:**
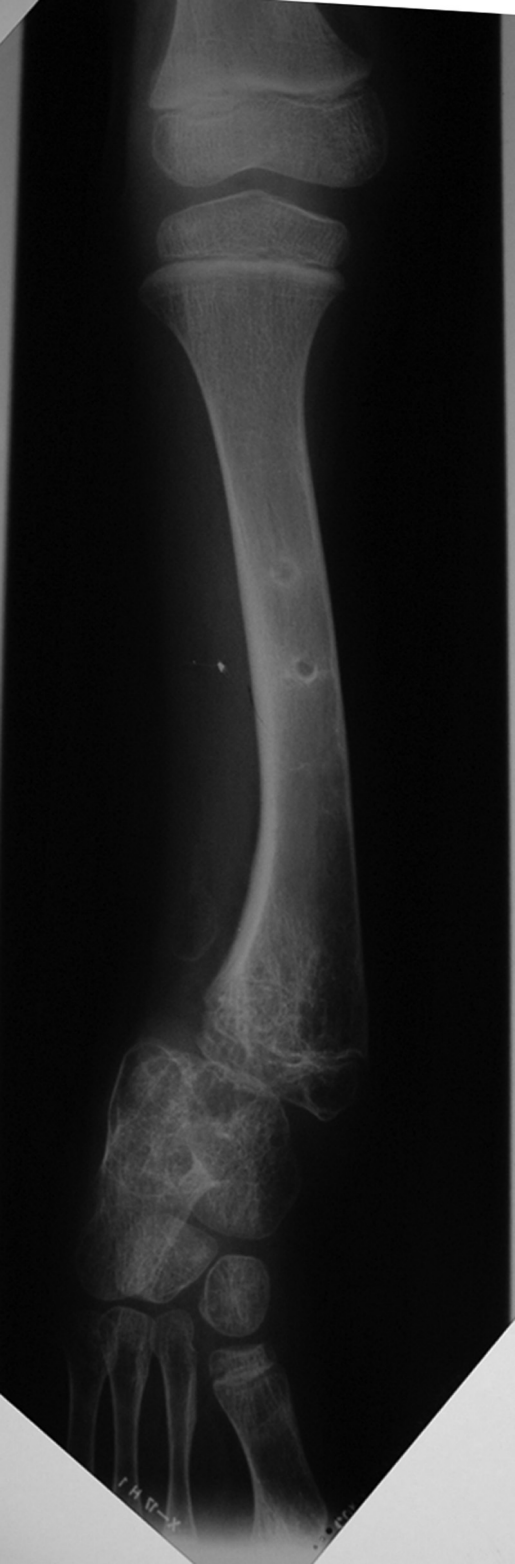
**Radiographic presentation of the same patient two years after new surgical intervention**. Radiograph of the same patient in the age of eleven years old and two years after new surgical intervention. The patient underwent a simultaneous closed corrective osteotomy and lengthening of the tibia. Thirteen months after completion of the consolidation phase there is a marked improvement although a distal tibia angulation of 12° still remains. Insufficient lateral buttress of the talus contributes to a valgus deformity of the ankle.

**Figure 4:**
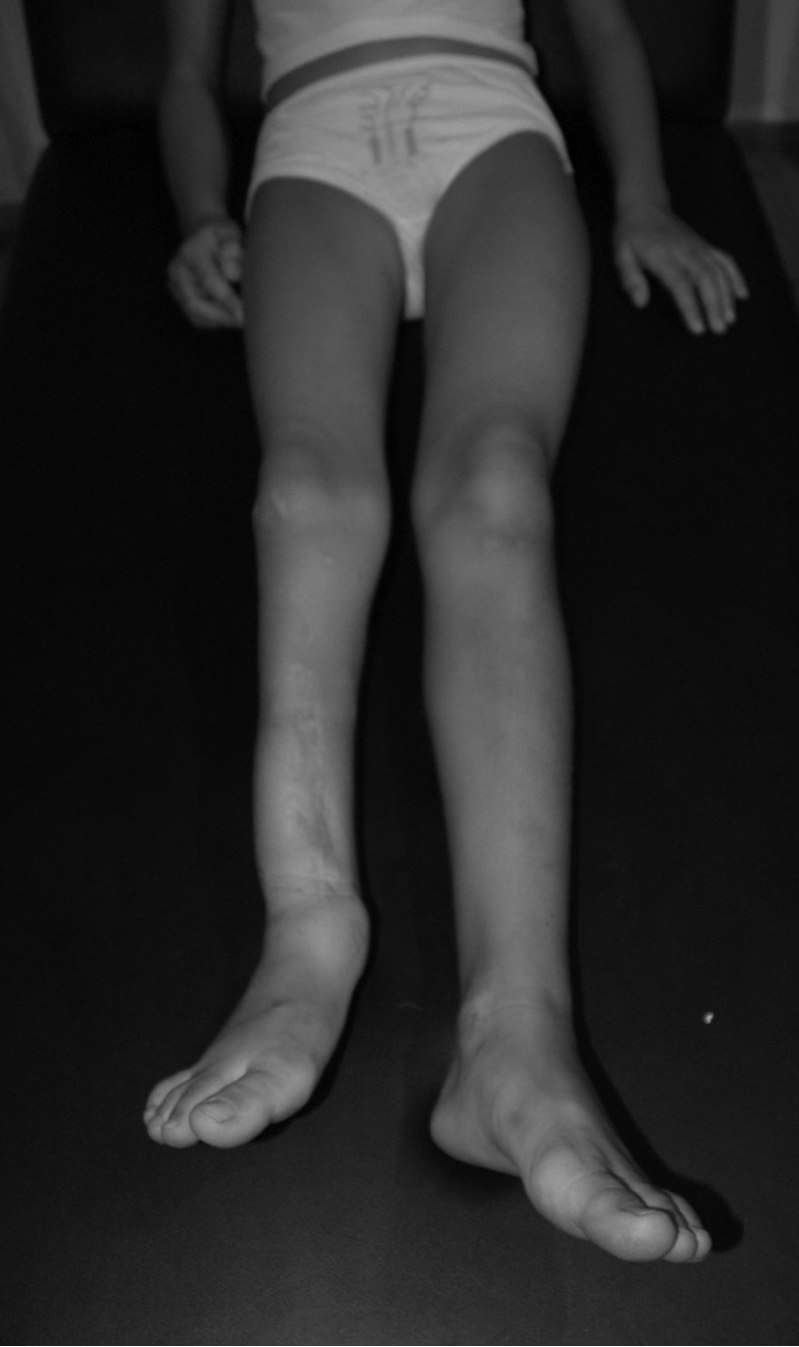
**Clinical presentation of the same patient in the age of thirteen years old**. Four years after completion of the second surgical procedure. Although there is an acceptable mechanical axis, there is a quite uncomfortable valgus deformity of the ankle. After two procedures, the overall length that was gained was only 3.0 cm in the cost of multiple previous efforts to correct axial malalignment in priority. Marked calf atrophy and a leg length discrepancy of 5.0 cm are clearly shown.

Five of seven patients [Additional file 2, table s2] presented even minimal limp after treatment, although limp by its own was not considered to severely impair functional ability of these patients (average LEFS 85.2%, ranged 53% to 98%). Residual anterior-medial bowing of the tibia was observed in four patients [Additional file 2, table s2]. One patient (case 6) [Additional file 1, table s1] presented twelve degrees of anterior and medial bowing of the tibia (significantly improved compared to 85 degrees of the initial deformity) (Figures 1, 2, 3, 4). Three patients presented less than five degrees of anterior-medial bowing of the tibia that has not been considered to cause functional problems, although one of them occasionally uses an in-shoe ankle cast due to valgus ankle. Calf atrophy was present in all seven patients and it was ipsilateral to fibular hemimelia. Strength of the involved muscles of the lower extremity was evaluated as normal despite calf atrophy. Valgus deformity of the ankle was present after treatment in two patients [Additional file 2, table s2]. Severe anterior-medial angulation of the tibia combined with hypoplasia of the tarsal bones was mainly responsible for the valgus ankle in one patient, who will probably need an additional corrective procedure in the future. Valgus deformity of the ankle and foot in one patient improved considerably after corrective osteotomy and lengthening of the tibia, although an inshoe cast is occasionally used. Hypoplasia or aplasia of certain foot rays with decreased longitudinal length of the ipsilateral foot was finally present in four patients [Additional file 2, table s2] being 1 cm, 1.5 cm, 0.5 cm and 3 cm respectively and did not consist in any patient a functional problem [Additional file 1, table s1], [Additional file 3, table s3].

Pin infection occurred in two patients and treated successfully with oral antibiotics. A painful hypertrophic non-union of the fibula at the osteotomy site was developed in one patient. Distraction at the osteotomy site of the tibia could not start in one patient who was needed to undergo a revision osteotomy on the 7^th ^post-operative day which followed by identical distraction of the osteotomized bone segments. There were no vascular and nerve injuries, no non-unions and no major medical complications.

## Discussion

In total or partial congenital absence of the fibula, various co-existing problems have to be addressed like leg length discrepancy, valgus deformities of the tibia and ankle joint, congenital deformities of the foot, and femoral hypoplasia [3].

Lengthening procedures have gained more popularity cause to recent advances in surgical techniques and better understanding of biologic and biomechanical features of fibular hemimelia. Lengthening of as much as 7 to 10 cm for the tibia and 16 cm for the femur has been achieved according to previous reports [6,12,13]. Published results are quite promising focusing on gained length and improvement of functional scores [11]. In previous series, a number of post-treatment complications have been reported [3,6]. In the present study, we tried to focus on the most frequent residual malformations and deformities after treatment of fibular hemimelia.

An average leg length discrepancy (LLD) of 4.08 cm was finally present after treatment in six of seven patients and in ages close to skeletal maturity. Concerning that one patient denied further lengthening procedures, true average post-treatment LLD could be re-calculated to 3.1 cm. This finding is in accordance to other series, where as other reports showed more promising results [3,6].

Three patients (cases 2, 3 and 6) [Additional file 1, table s1] tolerated very well discrepancies up to 4 cm for extended periods while waiting for the lengthening procedure. Ipsilateral calf atrophy was present in all patients, being more prominent in four patients who underwent lengthening procedures and in one patient after tarsal osteotomy. None of these patients complained for muscle weakness and functional disability. These findings were consistent with the results of Kaljumae et al. who hypothesized that lengthening of the femur could create an ischemic environment and result in muscle atrophy although they did not detect any functional impairment of the extremities [14]. Yoshipovitch and Palti had already proved pathological changes of blood pressure in the extremities during lengthening [15]. As calf atrophy considered a cosmetic problem for several patients, parents should be well informed about this issue.

Limp was present in five of seven patients, where as in only one patient (case 7) it was severe and consisted a functional problem. Patients with a considerable limp presented residual length discrepancy of more than 4 cm except one patient who although had a complete leg length equalization presented valgus deformity of the ankle. Valgus deformity of the ankle is quite common in patients with fibular hemimelia. This is especially true in total absence of the fibula, but even in milder forms the insufficient lateral buttress of the ankle results in progressive valgus deformity. Supramalleolar osteotomy has been proposed for moderate to severe forms, where as others have suggested screw epiphysiodesis of the medial malleolus [3]. This was not the case in our series of patients, and only two of the totally seven patients presented an obvious valgus deformity of the ankle after treatment. Both patients presented a type II aplasia of the fibula, whereas one of them presented severe dysplasia of the lateral part of the distal tibial epiphysis [16]. The latter has been suggested as a cause of resisting to treatment valgus deformity of the distal tibia and ankle [4]. One patient with mild dysplasia of the distal tibial epiphysis responded well to lengthening procedures without residual valgus deformity, which is in accordance to the conclusions of Choi et al. [4]. Both patients with a residual valgus ankle presented simultaneously persistent anterior-medial angulation of the tibia, an indication that these two deformities are strongly correlated. Two patients with milder forms of tibia angulation did not present a valgus ankle. In only one patient angulation of the tibia which was associated with severe dysplasia of the distal tibial epiphysis consisted a severe problem which required multiple procedures. It is still remaining in question if angulation of the tibia is impaired during and after lengthening procedures [17].

None of the three patients with foot rays aplasia or hypoplasia had functional problems because of this deformity. Stevens & Arms also concluded that none of the four patients of their series with foot rays aplasia/hypoplasia had functional problems [3]. Although the absence of foot rays (usually the lateral ones) is the most obvious deformity on birth it did not show to be disabling. Decreased longitudinal length of the foot up to three cm did also show to be of no clinical consequence and did not require a treatment, either if the shorter axis was due to congenital hypoplasia or impaired after tarsal closed osteotomy. Observed increased healing index although expected should be counted as an additional problem which tests patients' tolerance and patience.

## Conclusions

Although the number of patients is limited, we believe that the present study contributes to the knowledge of most expected residual deformities and malformations after treatment of fibular hemimelia. Despite improvements in surgical techniques and better understanding of the characteristics of this type of congenital deformity, a considerable number of persisting malformations seems to remain after treatment. Parents should have the right expectations and in this instance they will be more co-operative and have better understanding in case of additional required procedures. Fibular hemimelia is very often associated with several concurrent deformities and needs meticulous and sophisticated treatment planning. Better knowledge of the expected problems after initial treatment might be helpful in appropriate treatment planning and in gaining better final results.

## Competing interests

The authors declare that they have no competing interests.

## Authors' contributions

DAA conceived of the study, participated in acquisition, analysis and interpretation of the data, in drafting and critical revision of the manuscript, in its design and coordination. AVK participated in analysis and interpretation of the data, helped in the design of the study and contributed in drafting of the manuscript and in revising it critically. AEB helped in analysis and interpretation of the data, in the design of the study in drafting of the manuscript and in revising it critically. PNS helped in analysis and interpretation of the data, in the design of the study in drafting of the manuscript and in revising it critically. All authors read and approved the final manuscript.

## Authors' information

DAA is Consultant, Department of Orthopaedic Surgery, General Hospital of Giannitsa, Giannitsa, Greece. He is regularly involved in the field Paediatric Orthopaedics and his thesis was in "Congenital Deformities of the Lower Extremities".

AVK is Assistant Professor, Department of Orthopaedic Surgery, University of Ioannina, Ioannina, Greece. He is regularly involved in the fields of Paediatric Orthopaedics and he is an expertise in Microsurgery.

AEB is Professor, Department of Orthopaedic Surgery, University of Ioannina, Ioannina, Greece. He is an expertise in the fields of Paediatric Orthopaedics, Lengthening procedures and Microsurgery PNS is Professor, 1st Department of Orthopaedic Surgery, School of Medicine, University of Athens, "Attikon" Hospital, Rimini 1 Haidari 12462, Athens, Greece. He is an expertise in the fields of Paediatric Orthopaedics, Lengthening procedures and Microsurgery.

## Supplementary Material

Additional file 1 **Table 1**. Details of the patients. Details of the patients at initial presentation and of the types of treatment. *: Right. **: Left. ***: Type of fibular hemimelia according to the Achterman-Kalamchi classification system [16]. ****: Leg Length Discrepancy (LLD) at the initial presentation of the patient. *****: Hypoplasia of the tibia was additionally present as a concurrent congenital deformity in all of the patients. Click here for file

Additional file 2 **Table 2**. Residual malformations and leg length discrepancy. Residual malformations, problems and leg length discrepancy after treatment at the end of the follow-up. *: Right. **: Left. ***: Leg Length Discrepancy (LLD) after treatment at the end of the follow-up. Click here for file

Additional file 3 **Table 3**. Outcome evaluation. Functional scoring of the patients at the initial presentation and after treatment at the end of the follow-up. *: Lower Extremity Functional Scale (LEFS). Click here for file
