# Supplementary material for: Residual malformations and leg length discrepancy after treatment of fibular hemimelia
Source: J Orthop Surg Res. 2011 Sep 27;6:51. doi: 10.1186/1749-799X-6-51 (PMC3191474; doi:10.1186/1749-799X-6-51)
Supplement: Additional file 1 — Table 1. Details of the patients. Details of the patients at initial presentation and of the types of treatment. *: Right. **: Left. ***: Type of fibular hemimelia according to the Achterman-Kalamchi classification system [16]. ****: Leg Length Discrepancy (LLD) at the initial presentation of the patient. *****: Hypoplasia of the tibia was additionally present as a concurrent congenital deformity in all of the patients. [file 1749-799X-6-51-S1.DOC]

| Case | Sex | Leg  R*/L** | Type*** | LLD ****  (cm) | Concurrent  Deformities***** | Type of treatment |
| --- | --- | --- | --- | --- | --- | --- |
| 1 | M | L | IB | 5.2 | -Distal Tibial Epiphysis Dysplasia  -Foot Hypoplasia  -Femoral Hypoplasia | -Tibia Lengthening  -Achilles Lengthening |
| 2 | F | R | II | 5.2 | -Cong. Angulation of the tibia  -Femoral Hypoplasia  -Lateral Femoral Condyle hypoplasia | -Femoral Lengthening  -Tibia Lengthening  -Achilles Lengthening |
| 3 | M | R  L | II  IA | 3.6 | -Cong. angulation of the tibia  -Foot rays aplasia  -Tarsal coalition  -severe pes planovalgus  -Lateral Femoral Condyle hypoplasia | -Tarsus corrective osteotomy  -Peroneal lengthening  -Achilles Lengthening |
| 4 | M | R | II | 5.9 | -Cong. angulation of the tibia  -Foot rays aplasia  -Severe pes planovalgus  -Femoral Hypoplasia  -Hand Syndactyly | -Tibia Lengthening  -Achilles Lengthening  -Peroneal lengthening |
| 5 | F | L | IB | - | -Cong. angulation of the tibia  - Calcaneovalgus foot  -Foot Hypoplasia | Modified Shoe |
| 6 | F | R | II | 2.9 | -Cong. angulation of the tibia  - Foot rays hypoplasia  -Foot hypoplasia  -Foot Syndactyly | -Tibia Lengthening  -Tibia corrective ostetotomy  -Achilles Lengthening |
| 7 | F | L | II | 9.0 | -Femoral Hypoplasia  - Congenital Hip Dislocation | -Tibia Lengthening  -Femoral Lengthening |
