# Supplementary material for: Residual malformations and leg length discrepancy after treatment of fibular hemimelia
Source: J Orthop Surg Res. 2011 Sep 27;6:51. doi: 10.1186/1749-799X-6-51 (PMC3191474; doi:10.1186/1749-799X-6-51)
Supplement: Additional file 2 — Table 2. Residual malformations and leg length discrepancy. Residual malformations, problems and leg length discrepancy after treatment at the end of the follow-up. *: Right. **: Left. ***: Leg Length Discrepancy (LLD) after treatment at the end of the follow-up. [file 1749-799X-6-51-S2.DOC]

| Case | Leg  R*/L** | Post –treatmentLLD***  (cm) | Limp | Bowing of the tibia | Calf atrophy | Valgus ankle | Foot Hypoplasia | Foot rays aplasia/ hypoplasia |
| --- | --- | --- | --- | --- | --- | --- | --- | --- |
| 1 | L | 1.5 | No | Yes | Yes | No | Yes | No |
| 2 | R | 4.7 | Yes | No | Yes | No | No | No |
| 3 | R  L | 4.3 | Yes | Yes | Yes | No | Yes | Yes |
| 4 | R | 0.0 | Yes | Yes | Yes | Yes | No | Yes |
| 5 | L | 2.0 | No | No | Yes | No | Yes | No |
| 6 | R | 5.0 | Yes | Yes | Yes | Yes | Yes | Yes |
| 7 | L | 9.0 | Yes | No | Yes | No | No | No |
