# Supplementary material for: Residual malformations and leg length discrepancy after treatment of fibular hemimelia
Source: J Orthop Surg Res. 2011 Sep 27;6:51. doi: 10.1186/1749-799X-6-51 (PMC3191474; doi:10.1186/1749-799X-6-51)
Supplement: Additional file 3 — Table 3. Outcome evaluation. Functional scoring of the patients at the initial presentation and after treatment at the end of the follow-up. *: Lower Extremity Functional Scale (LEFS). [file 1749-799X-6-51-S3.DOC]

| Case | LEFS* pre-treatment  (% percentage) | LEFS post-treatment  (% percentage) |
| --- | --- | --- |
| 1 | 74 | 100 |
| 2 | 68 | 83 |
| 3 | 71 | 98 |
| 4 | 68 | 98 |
| 5 | 96 | 100 |
| 6 | 66 | 94 |
| 7 | 53 | 53 |
